# Dendritic Nanostructured Waste Copper Wires for High-Energy Alkaline Battery

**DOI:** 10.1007/s40820-019-0337-2

**Published:** 2019-12-12

**Authors:** Nilesh R. Chodankar, Su-Hyeon Ji, Young-Kyu Han, Do-Heyoung Kim

**Affiliations:** 1 School of Chemical Engineering, Chonnam National University, Gwangju, 500-757 South Korea; 2 Department of Energy and Materials Engineering, Dongguk University, Seoul, 100-715 Republic of Korea

**Keywords:** Alkaline batteries, Dendritic nanostructure, NiCo-hydroxide, Waste Cu wires

## Abstract

Rechargeable alkaline batteries (RABs) have received remarkable attention in the past decade for their high energy, low cost, safe operation, facile manufacture, and eco-friendly nature. To date, expensive electrode materials and current collectors were predominantly applied for RABs, which have limited their real-world efficacy. In the present work, we propose a scalable process to utilize electronic waste (e-waste) Cu wires as a cost-effective current collector for high-energy wire-type RABs. Initially, the vertically aligned CuO nanowires were prepared over the waste Cu wires via in situ alkaline corrosion. Then, both atomic-layer-deposited NiO and NiCo-hydroxide were applied to the CuO nanowires to form a uniform dendritic-structured NiCo-hydroxide/NiO/CuO/Cu electrode. When the prepared dendritic-structured electrode was applied to the RAB, it showed excellent electrochemical features, namely high-energy-density (82.42 Wh kg^−1^), excellent specific capacity (219 mAh g^−1^), and long-term cycling stability (94% capacity retention over 5000 cycles). The presented approach and material meet the requirements of a cost-effective, abundant, and highly efficient electrode for advanced eco-friendly RABs. More importantly, the present method provides an efficient path to recycle e-waste for value-added energy storage applications.

**Electronic supplementary material:**

The online version of this article (10.1007/s40820-019-0337-2) contains supplementary material, which is available to authorized users.

## Highlights

- Electronic waste Cu wires were successfully used as a cost-effective current collector for high-energy wire-type rechargeable alkaline batteries.
- The scalable approach was applied to reduce, reuse, and recycle electronic waste.
- A developed wire-type rechargeable alkaline battery exhibited a high-energy-density of 82.42 Wh kg^−1^ with long-term cycling stability.

**Electronic supplementary material:**

The online version of this article (10.1007/s40820-019-0337-2) contains supplementary material, which is available to authorized users.

## Graphical abstract

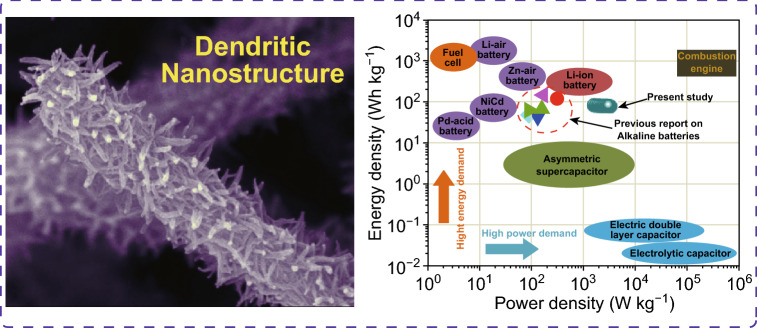

## Introduction

The extensive growth of portable electronics, implantable biomedical devices, and hybrid electrical vehicles demands low-cost, high-performance energy storage devices [1]. In the last two decades, lithium-ion batteries (LIBs) and supercapacitors (SCs) have been the dominant energy storage devices for portable and grid-level applications [2–5]. The higher energy density of LIBs makes them suitable for commercial application; however, the lower power capability, limited cycling stability, high cost, flammability, and toxicity of LIBs have limited their practical utility. In contrast, SCs have attracted immense interest for their higher power capability, long cycle life, and nontoxic nature. However, the surface charge storage mechanism of SCs leads to lower energy density and capacitance values [6]. For certain applications, energy storage devices must exhibit energy densities similar to those of LIBs and power capabilities similar to those of SCs. Recently, rechargeable alkaline batteries (RABs) have been considered as alternative energy storage devices for LIBs and SCs as they show higher energy density than SCs and higher power density than LIBs [7, 8]. Unlike the conventional liquid electrolyte-based planar RABs, wire-type solid-state RABs have recently attracted increased research interest owing to their flexible and lightweight nature, which is suitable for wearable electronics [9–11]. In addition to their flexible and lightweight nature, the lower energy storage capacity due to the smaller operating voltage window and limited cycling stability of wire-type RABs have inspired us to investigate advanced electrode materials, current collectors, and electrolytes to achieve higher energy density and cycling stability. In the literature, various electrode materials were reported to enable RABs to attain higher electrochemical performance. In particular, multicomponent electrode materials in the core–shell form are preferred to obtain higher energy storage capacities due to their synergistic effect [12, 13]. Nickel cobalt-based nanostructured electrode materials have attracted interest for their high abundance, high capacity, and good reversibility over several electrochemical cycles. In addition, the higher electrical conductivity and redox activity of nickel cobalt-based electrode materials make them suitable candidates for energy storage applications [14, 15]. Apart from electrode materials, identifying a low-cost current collector is also essential to minimize the production cost of energy storage devices.

The rapidly increasing human population, growing economy, rapid urbanization, and increase in living standards have greatly accelerated the rate of waste generation, thereby directly affecting the earth’s atmosphere. Most of this waste is toxic and contains hazardous and health-threatening chemicals. Proper waste management is the best way to maintain a clean atmosphere by reducing, reusing, and recycling our waste [16, 17]. Among the different types of waste, electronic waste (e-waste) is of serious concern to society and arises from discarding electronic equipment after the end of its useful life. The rapid increase in the demand for advanced electronic devices has led to the constant generation of enormous amounts of e-waste, thereby causing environmental issues, as they contain harmful materials such as lead, cadmium, and beryllium. Electric wires are a universally occurring form of e-waste and primarily contain metallic Cu or Al. Recycling these metallic waste wires for different energy applications will effectively mitigate economic and environmental concerns [18–22]. To develop an efficient energy storage device that is also cost-effective, it is imperative to search for a common material that could be employed as a current collector, active material, and electrolyte for suitable expanse, as well as possesses adequate energy storage capacity [23, 24]. In general, various current collectors have been used to develop energy storage devices, including stainless steel, carbon cloth, Ti foil, and Ni foam [23]. However, these current collectors are expensive, which will increase the production cost of the energy storage device. To control the production cost, the utilization of waste Cu wires as a current collector to assemble energy storage devices is the best option.

With this motivation, in the present work, we have developed a wire-type RAB using waste Cu wires as a current collector. Briefly, a dendritic-structured NiCo-hydroxide/NiO/CuO/Cu electrode has been designed by combining wet (alkaline corrosion and chemical bath deposition) and dry (atomic-layer deposition) processes, to yield a high-specific-capacity electrode for batteries, excellent rate capability, and long-term cycling performance when used in an RAB. The designed electrode provides sufficient interspace, as well as a multichannel pathway, for the electrolyte penetration to enable efficient charge and mass transfer within the bulk materials. Moreover, the dendritic-structured electrode prepared over the waste Cu wire itself acts as a self-supportive electrode to control the resultant resistance of the electrode. This synthesis route also provides the facial route to utilize the cost-effective current collector for energy storage applications [25, 26].

## Experimental Section

### Materials and Chemicals

All chemicals were used as received from Sigma-Aldrich Corp., South Korea. The copper (Cu) wires used in the experiments were collected from our department’s storeroom. All chemicals were analytical grade and used as received without any further processing. All precursor solutions were prepared in deionized water.

### Preparation of Cu(OH)_2_ Nanowires on Cu Wire

Prior to the preparation of Cu(OH)_2_ nanowires on the Cu wire, the plastic coating of the scrap Cu wire was removed by a wire stripper, and then the obtained Cu fibers were braided to form a single structure. Furthermore, the Cu wire was cleaned with 1 M HCl and deionized (DI) water to remove the native oxide layer from its surface. To grow the Cu(OH)_2_ nanowires, the typical alkaline corrosion method was used. The precursor solution was prepared by dissolving (NH_4_)_2_S_2_O_8_ (4.107 g) and NaOH (11.997 g) in 100 mL of DI water with continuous stirring for 30 min. The cleaned Cu fibers were then prepared at a length of 5 cm and kept in the prepared solution for 5 min to form the Cu(OH)_2_ nanowires. The active region of the electrode occupied 4 cm of the wire, and the remaining 1 cm, was used for the electric contacts. During the reaction time, the color of the solution changed from transparent to blue indicating the formation of Cu(OH)_2_ nanowires over waste Cu wires. Furthermore, the prepared Cu(OH)_2_/Cu wire sample was rinsed in DI water and kept at 60 °C overnight.

### Preparation of Thin NiO on the Cu(OH)_2_/Cu Wire

To enhance the electrical conductivity and surface area of the Cu(OH)_2_/Cu wire, thin NiO was carried out with a homemade atomic-layer deposition (ALD) system. Commercially available Ni(EtCp)_2_ was used as the precursor with O_2_ plasma as the oxidant. The temperature of the precursor was maintained at 50 °C with a line temperature of 60 °C, and the deposition chamber was maintained at 250 °C. Initially, the ALD conditions were optimized by conducting the deposition of NiO over a silicon substrate. The standard optimized conditions for the NiO ALD process were as follows: precursor pulsing (30 sccm, 1.5 s), main purging (30 sccm, 20 s), bypass purging (250 sccm, 5 s), oxygen plasma (5 s), and Ar purging (30 s). Under these conditions, the growth rate of the NiO layer was 0.037 nm per cycle at 250 °C. After the ALD parameters were optimized, NiO was deposited over the Cu(OH)_2_/Cu nanowires for 200 cycles.

### Preparation of NiCo-Hydroxide/NiO/CuO/Cu Electrode

The nanowires of NiCo-hydroxide were prepared on the NiO/CuO/Cu electrode via low-temperature chemical bath deposition. The precursor solution was prepared by dissolving 0.05 M Co(NO_3_)_2_·H_2_O, 0.05 M Ni(NO_3_)_2_·6H_2_O, and 0.25 M urea in 50 mL DI water. Afterward, the NiO/CuO/Cu electrode was immersed in the precursor solution and kept in the oven at 80 °C for 2 h to form the NiCo-hydroxide nanowires over the NiO/CuO/Cu electrode. After the growth process, the sample was removed from the precursor solution, then rinsed with DI water, and dried in the oven at 60 °C overnight. The mass loading of the material (NiO and NiCo-hydroxide) over CuO/Cu is 0.56 mg (0.14 mg cm^−1^). The prepared dendritic-type NiCo-hydroxide/NiO/Cu(OH)_2_/Cu electrode was used to assemble the wire-type rechargeable aqueous battery.

### Preparation of Gel Electrolyte and Wire-Type Rechargeable Aqueous Battery

The PVA–KOH gel electrolyte was prepared by mixing 2 M KOH and 2 g of PVA in 20 mL of DI water at 70 °C for 20 min while stirring. The formed transparent gel-like solution was used to assemble the wire-type rechargeable aqueous battery by immersing both electrodes (4 cm), i.e., NiCo-hydroxide/NiO/CuO/Cu and AC/SS, in the PVA–KOH gel electrolyte for 20 min to cover all the active sites of the material with the gel electrolyte, which was then hung in the oven at 60 °C for 6 h to remove the water content from the gel electrolyte. After that, both electrodes were held together and again dipped in the gel electrolyte and solidified at 60 °C for 6 h. Finally, the assembled device was covered with parafilm and used for further electrochemical measurements. The mass ratio of the positive to negative electrode was determined by the well-known charge-balance equation (*q*+ = *q*−) to be 1:4.5.

### Preparation of Activated Carbon/Stainless Steel Electrode

To assemble the full rechargeable aqueous battery, a negative electrode is required, whereas NiCo-hydroxide/NiO/CuO/Cu will act as a positive electrode. To prepare the negative electrode, the stainless steel wires were collected from the 200 stainless steel mesh (304 type). The 20 stainless steel wires were braided to form a uniform structure. The traditional activated carbon coating was carried out over the braided stainless steel wires. Typically, a uniform mixture of the activated carbon (80%), carbon black (10%), and PVDF (10%) with a few drops of ethanol was prepared and loaded over the braided stainless steel wires. To enhance the compatibility between the activated carbon and stainless steel wires, the prepared electrode was heated on a hot plate for 1 h at 200 °C.

### Electrochemical Measurements

The electrochemical measurements for the prepared electrode and wire-type rechargeable aqueous battery were carried out by performing cyclic voltammetry (CV), galvanostatic charge–discharge (GCD), and electrochemical impedance spectroscopy (EIS). For the three-electrode measurements, electrochemical measurements were carried out using the NiCo-hydroxide/NiO/CuO/Cu electrode as a working electrode, platinum plate as a counter electrode, and saturated calomel electrode (SCE) as the reference electrode in 2 M potassium hydroxide (KOH). The two-electrode measurements were performed by fabricating a wire-type rechargeable aqueous battery with PVA–KOH gel electrolytes.

The thickness of the ALD films was measured by ellipsometry (Gaertner Scientific). The surface morphology of the prepared samples was characterized using field-emission scanning electron microscopy (FE-SEM, JEOL JSM-7500F) and high-resolution transmission electron microscopy (HR-TEM, JEOL JEM-2100F). The crystal structure and oxidation state of the prepared samples were confirmed by X-ray diffraction (XRD, X’Pert Pro using CuKg radiation) and X-ray photoelectron spectroscopy (XPS, ESCALAB-MKII).

The electrochemical parameters include the specific capacitance (F g^−1^), length capacitance (mF cm^−1^), and specific capacity (mAh g^−1^), which were calculated by the following equations:

$${\text{Specific}}\,{\text{capacitance}}{:}\,Cs = I\Delta t /\left( {m\Delta V} \right)$$

$${\text{Length}}\,{\text{capacitance}}{:}\,CL = I\Delta t /\left( {L\Delta V} \right)$$

$${\text{Specific}}\,{\text{capacity}}{:}\,Q = I\Delta t /\left( {3.6\,{\text{m}}} \right)$$

here *I*(A) is the discharge current, ∆*t* (s) is the discharge time, *m* (g) is the mass of the active material, ∆*V* (V) is the potential, and *L* (cm) is the length of the electrode/device.

## Results and Discussion

**Fig. 1:**
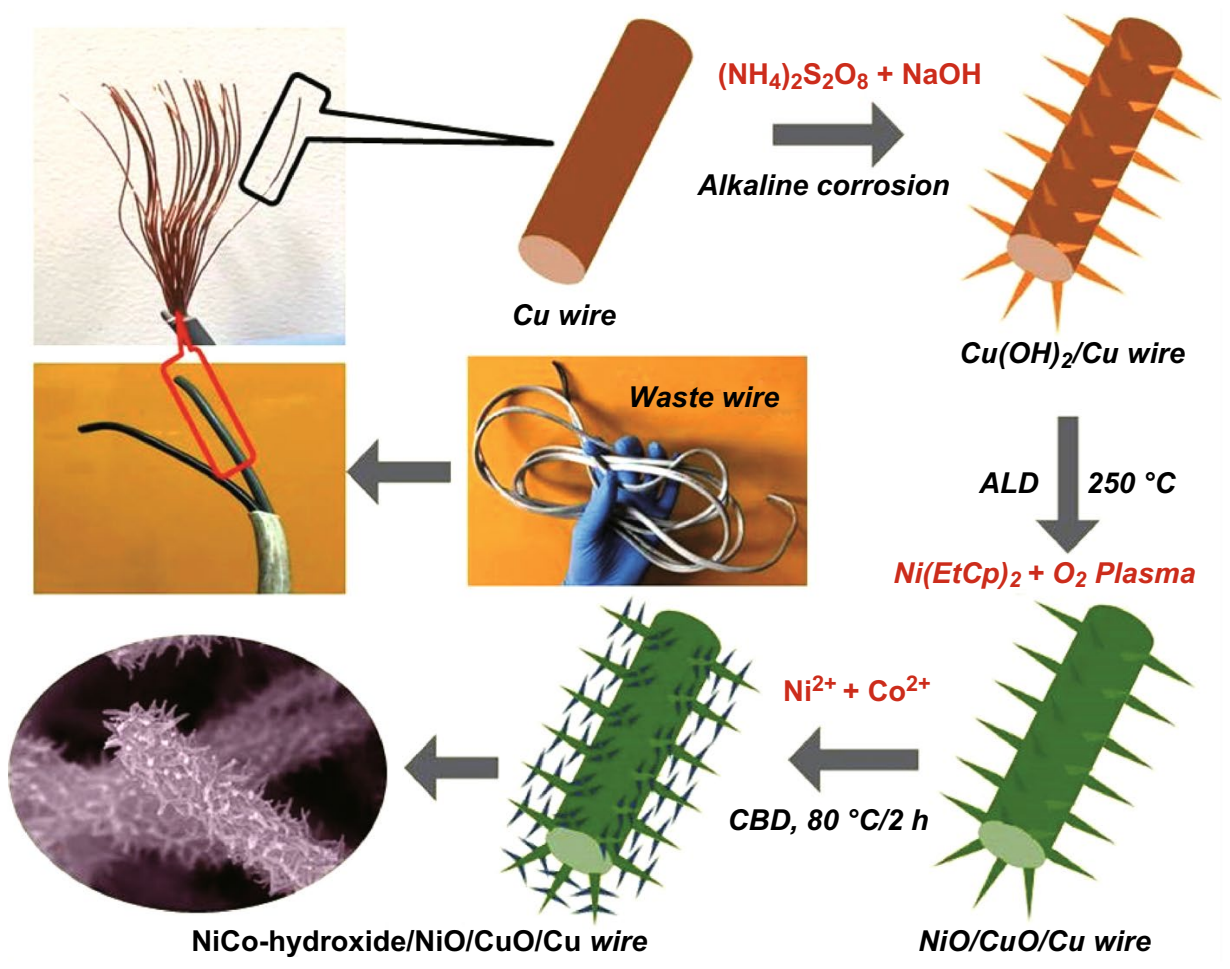
Schematic illustration of the preparation of dendritic-structured NiCo-hydroxide/NiO/CuO/Cu electrode using e-waste Cu wires for the RAB application

The stepwise preparation of the dendritic-structured NiCo-hydroxide/NiO/CuO/Cu electrode is presented in Fig. 1. Waste management is critical to maintain a clean atmosphere for living creatures. With this motivation, and to minimize e-waste, we used e-waste Cu wires for energy storage applications. The fabrication of the electrode involves three major steps. First, the plastic nonconducting coating of the waste Cu wires was removed by wire stepper, and the obtained Cu wires were treated with an alkaline solution for a very short time (5 min) to form vertically aligned Cu(OH)_2_ nanowires. However, the lower electrical conductivity of the Cu(OH)_2_ is a major obstacle for facial electrochemical reactions [27]. Second, to enhance the electrical conductivity of the Cu(OH)_2_ nanowires, ALD of NiO film (200 cycles) was carried out on the Cu(OH)_2_ nanowires. Here, ALD NiO plays multiple roles in enhancing the energy storage capacity of the RAB and enhances the electrical conductivity of the electrode to increase the power capability of the RAB. Furthermore, it will contribute to the redox capacity and allow more active sites to host NiCo-hydroxide. As the ALD of NiO film was carried out at a reaction temperature of 250 °C, Cu(OH)_2_ was converted into CuO, which will, again, be beneficial in enhancing the electrochemical features of the RAB. Finally, the NiCo-hydroxide nanowires were prepared over the NiO/CuO/Cu wires by conventional chemical bath deposition (CBD) at a low reaction temperature of 80 °C to form the dendritic-structured NiCo-hydroxide/NiO/CuO/Cu electrode.

**Fig. 2:**
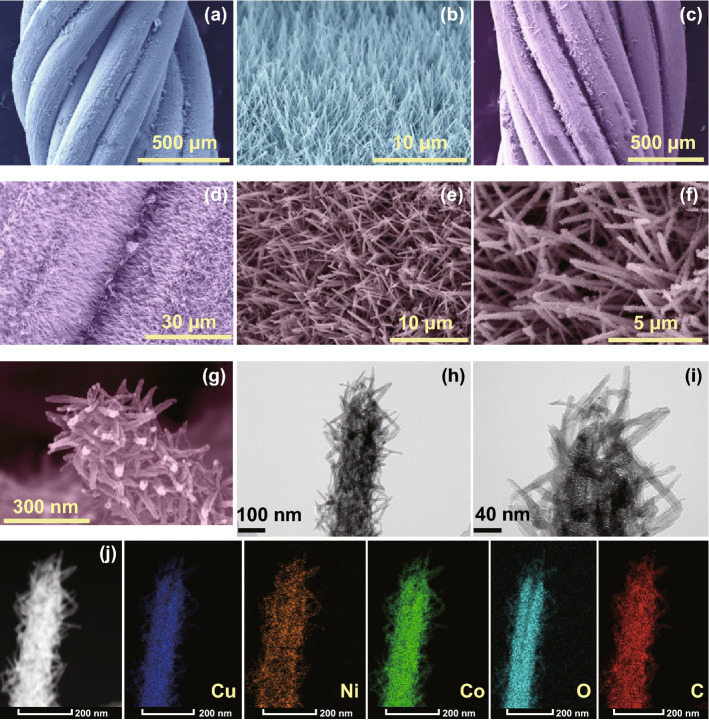
FE-SEM images for the **a**, **b** Cu(OH)_2_/Cu wire, **c**, **d** NiO/CuO/Cu, and **e–g** NiCo-hydroxide/NiO/CuO/Cu electrodes. **h**, **i** TEM images for the NiCo-hydroxide/NiO/CuO/Cu electrode. **j** STEM-EDS elemental mapping results for the NiCo-hydroxide/NiO/CuO/Cu electrode

The physical and chemical properties of the as-prepared dendritic-structured NiCo-hydroxide/NiO/CuO/Cu electrode were determined by X-ray diffraction (XRD), X-ray photoelectron spectroscopy (XPS), field-emission scanning electron microscopy (FE-SEM), and transmission electron microscopy (TEM) measurements to verify that the quality of the materials could meet the requirements for RAB applications. Figure 2 shows the FE-SEM and TEM results for the prepared electrodes. As shown in Fig. 2a, b, Cu(OH)_2_ nanowires are layered uniformly over the Cu wire after alkaline corrosion. The vertically aligned and well-separated Cu(OH)_2_ nanowires are beneficial for energy storage applications, as they provide sufficient interspacing for ion movement. However, to overcome the limited conductivity of the Cu(OH)_2_ nanowires, thin NiO deposition (200 cycles) is carried out by ALD. Even after 200 ALD NiO cycles, the electrode still maintained basic nanowire-like nanostructures due to the conformal deposition in the ALD technique, as well as maintained its macroscopic nature without blocking the macropores, as shown in Fig. 2c, d [28–30]. TEM analysis for the NiO/CuO/Cu electrode is presented in Fig. S1, which clearly shows the formation of a very thin (~ 7 nm) NiO coating over the CuO/Cu nanowire electrode. The surface morphology of the NiCo-hydroxide/NiO/CuO/Cu electrode is presented in Fig. 2e–g at different magnifications. The low-magnification images clearly show the formation of the dendritic nanostructures through the surface of the sample (Fig. 2e, f), whereas the high-magnification SEM image shows the formation of very fine and vertically aligned NiCo-hydroxide nanowires over NiO/CuO/Cu to form dendritic nanostructures. TEM images also show the formation of dendritic nanostructures for the NiCo-hydroxide/NiO/CuO/Cu electrode (Fig. 2h, i). After CBD of the NiCo-hydroxide, each NiO/CuO/Cu nanowire is decorated with NiCo-hydroxide nanowires to form a branched nanostructure, which will enhance the energy storage capacity of the RAB by providing a large surface area as well as redox capacity [31, 32]. From the energy storage perspective, this type of nanostructure is favorable for charge storage as it provides a large surface area as well as multiple channels for charge transfer that will simultaneously boost the energy and power densities. Moreover, each dendritic nanowire is separated from the others by the formation of a porous nanostructure, which will provide large and open channels for the diffusion of electrolyte ions. Scanning transmission electron microscopy (STEM)–energy-dispersive X-ray (EDX) elemental mapping for the NiCo-hydroxide/NiO/CuO/Cu electrode is shown in Fig. 2j, which confirms the uniform distribution of each element through the surface of the electrode. The phase and surface chemical state of the prepared electrodes were measured by XRD and XPS, and the corresponding results are presented in Figs. S2, S3.

**Fig. 3:**
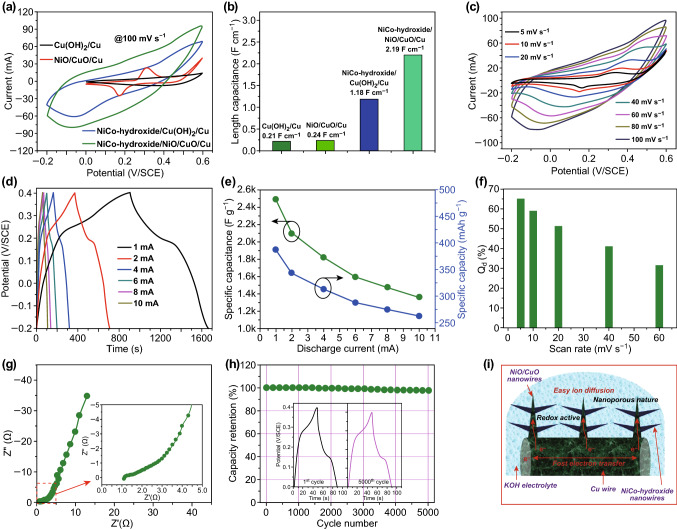
**a** Comparative CV curves and **b** corresponding length capacitance for the Cu(OH)_2_/Cu, NiO/CuO/Cu, NiCo-hydroxide/Cu(OH)_2_/Cu and NiCo-hydroxide/NiO/CuO/Cu electrodes at a constant scan rate of 100 mV s^−1^. **c** CV and **d** CD curves for the NiCo-hydroxide/NiO/CuO/Cu electrode at various scanning rates in 2 M KOH electrolyte. **e** specific capacitance and specific capacity for the NiCo-hydroxide/NiO/CuO/Cu electrode at various currents. **f** Plot of diffusion-controlled contribution at different scan rates for the NiCo-hydroxide/NiO/CuO/Cu electrode. **g** Nyquist plot for the NiCo-hydroxide/NiO/CuO/Cu electrode. The inset shows the magnified view of the Nyquist plot. **h** Plot of capacity retention versus cycle number for the NiCo-hydroxide/NiO/CuO/Cu electrode. The inset shows the first and last cycles. **i** Schematic representation showing the merits of the NiCo-hydroxide/NiO/CuO/Cu electrode for the RAB application

To demonstrate the electrochemical superiority of the NiCo-hydroxide/NiO/CuO/Cu electrode for the RAB, comparative cyclic voltammetry (CV) measurements were carried out in a three-electrode system with 2 M KOH electrolyte. Figure 3a shows the comparative CV curves for all the electrodes at an identical scan rate of 100 mV s^−1^, indicating a higher integral area with large anodic and cathodic currents for the NiCo-hydroxide/NiO/CuO/Cu electrode, thus suggesting a higher energy storage capacity [33]. To prove our claim, we calculated the length capacitance for all the electrodes and plotted them in Fig. 3b. The higher length capacitance of the NiCo-hydroxide/NiO/CuO/Cu electrode (2.19 F cm^−1^) is indicative of its improved electrochemical features compared to the other electrodes. Furthermore, to determine the rate capability of the NiCo-hydroxide/NiO/CuO/Cu electrode, CV and charge–discharge (CD) measurements were carried out at various scanning rates, and the corresponding results are presented in Fig. 3c, d. The CV curves show nonrectangular behavior, with a pair of redox peaks indicating the faradaic redox processes of the electrode components. More importantly, the CV curves maintain their shape at both lower and higher scanning rates, signifying better rate capability [34, 35]. Figure 3d shows the representative CD curves for the NiCo-hydroxide/NiO/CuO/Cu electrode at different currents ranging from 1 to 10 mA. The CD curves show nonlinear behavior, suggesting the existence of faradaic processes, and are consistent with the CV results. The CD curves are approximately symmetric in nature, without any potential drop, even at a higher current of 10 mA, indicating that the dendritic NiCo-hydroxide/NiO/CuO/Cu electrode has good electrochemical characteristics and superior reversible redox properties. The discharge-specific capacitance and specific capacity were calculated for the NiCo-hydroxide/NiO/CuO/Cu electrode at various currents and are plotted in Fig. 3d. At a low current of 1 mA, the electrode shows the highest specific capacity of 387.37 mAh g^−1^ (2486 F g^−1^), which decreases to 262.62 mAh g^−1^ (1363.63 F g^−1^) at a higher current of 10 mA with a rate capability of 67.79%. This suggests that the developed electrode has better electrochemical characteristics. Furthermore, we quantified the capacitive (*Q*_c_) and diffusion (*Q*_d_) controlled contribution to the overall current response in the CV curves for the NiCo-hydroxide/NiO/CuO/Cu electrode, and the obtained results are presented in Fig. 3f. The diffusion-controlled contribution is dominant at low scan rates, whereas the capacitive contribution is higher at a high scan rate. At a low scan rate of 5 mV s^−1^, the diffusion-controlled contribution is ~ 65%, indicating the facial diffusion of the electrolyte ions in the active electrode materials for the faradaic reactions. With an increase in scan rate from 5 to 60 mV s^−1^, it is reasonable to observe that the diffusion-controlled contribution decreases to ~ 32%, signifying that surface redox reactions dominate at the high scan rate.

To further understand the electrochemical kinetics of the NiCo-hydroxide/NiO/CuO/Cu electrode, EIS was carried out in the frequency range of 100 kHz to 10 MHz. Figure 3g shows the obtained Nyquist plot. In order of decreasing frequency, the first intercept of the Nyquist plot in the high-frequency region shows the equivalent series resistance (*R*_s_), the diameter of the semicircle in the high-frequency region provides the charge transfer resistance (*R*_ct_), and the vertical line in the low-frequency region represents the electrolyte ion diffusion in the structure of the electrode materials [36, 37]. In the present case, the NiCo-hydroxide/NiO/CuO/Cu electrode shows a lower *R*_s_ (1.07 Ω) as well as *R*_ct_ (0.32 Ω), suggesting a facial electrochemical reaction between the electrolyte ions and active electrode materials. The long-term cycling stability is, again, an important factor for energy storage devices. Here, the cycling stability for the NiCo-hydroxide/NiO/CuO/Cu electrode was measured by performing CD measurements at a high current of 12 mA for 5000 cycles, where a 97% capacity retention is observed. The excellent electrochemical performance in terms of higher specific capacity, rate capability, and long-term cycling stability for the dendritic-structured NiCo-hydroxide/NiO/CuO/Cu electrode is mainly due to its hierarchical nanoporous structure, as schematically presented in Fig. 3i. First, the vertically aligned CuO nanowires themselves act as a current collector that will drastically reduce the resultant resistance of the electrode. Second, the vertically aligned CuO nanowires provide a large interspace for electrolyte penetration, as well as to host the NiO and NiCo-hydroxide [38, 39]. Third, the ALD NiO over the CuO nanowires enhances the electrical conductivity, resultant surface area, redox sites for the electrochemical reactions, and NiCo-hydroxide hosting [40]. Fourth, the NiCo-hydroxide nanowires over the NiO/CuO/Cu nanowires drastically enhance the electroactive surface area for the redox reactions to enhance the energy storage capacity of the electrode. The interconnected arrangement of the NiCo-hydroxide and NiO/CuO/Cu nanowires creates abundant pathways for electrolyte penetration. The amorphous nature of NiCo-OH is favorable for charge storage because it provides abundant grain boundaries and ion diffusion channels. In addition, the binderless approach avoids unnecessary dead surface area by enhancing the electrical conductivity of the electrode [41–43]. Finally, in addition to the synergetic effect of the dendritic structure, the low cost and abundant availability of the waste Cu wires with low-cost processing make them strong candidates for energy storage applications.

**Fig. 4:**
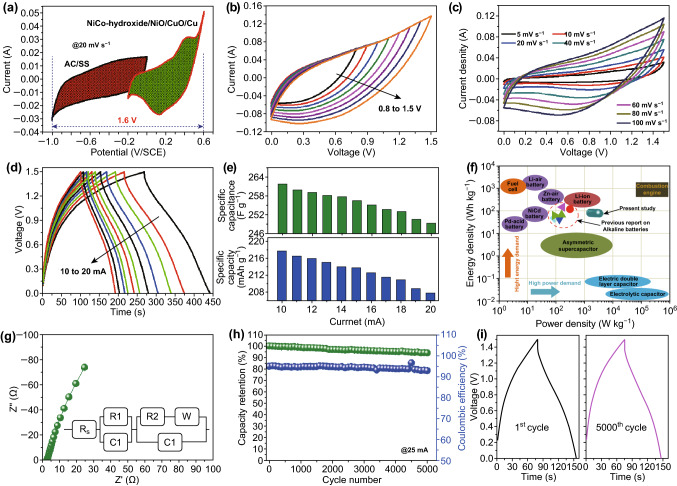
CV curves for **a** the AC/SS and NiCo-hydroxide/NiO/CuO/Cu electrode in 2 KOH electrolyte at a constant scan rate of 20 mV s^−1^, **b** the NiCo-hydroxide/NiO/CuO/Cu//AC/SS RAB at various applied voltage ranges, and **c** the NiCo-hydroxide/NiO/CuO/Cu//AC/SS RAB at various scan rates in the voltage window of 0–1.5 V. **d** CD curves for the NiCo-hydroxide/NiO/CuO/Cu//AC/SS RAB at various currents. **e** Plot of specific capacitance and specific capacity at various discharge current for the NiCo-hydroxide/NiO/CuO/Cu//AC/SS RAB. **f** Ragone plot for the NiCo-hydroxide/NiO/CuO/Cu//AC/SS RAB. **g** Nyquist plot for the NiCo-hydroxide/NiO/CuO/Cu//AC/SS RAB; the inset shows the fitted equivalent circuit. **h** Plot of capacity retention and coulombic efficiency for the NiCo-hydroxide/NiO/CuO/Cu//AC/SS RAB; and **i** initial and final CD cycles at a constant current of 25 mA

To demonstrate the actual application of the prepared electrode to batteries, an RAB was assembled with NiCo-hydroxide/NiO/CuO/Cu as a positive electrode and activated carbon (AC)/stainless steel (SS) as a negative electrode with aqueous PVA-KOH gel electrolyte. Figure 4a shows the CV curves for the NiCo-hydroxide/NiO/CuO/Cu and AC/SS electrode in the three-electrode system at a constant scan rate of 20 mV s^−1^ in 2 M KOH electrolyte. As observed in the CV curve, the NiCo-hydroxide/NiO/CuO/Cu electrode can work in the positive potential (− 0.2 to 0.6 V/SCE), whereas the AC/SS electrode shows good electrochemical performance in the negative potential (− 1.0 to 0 V/SCE); therefore, it is possible to combine these electrodes in a single cell to enhance the energy storage capacity and operating voltage of the RAB. To optimize the voltage window for the assembled NiCo-hydroxide/NiO/CuO/Cu//AC/SS RAB, the CV measurements were carried out at various voltage windows (Fig. 4b). The optimized voltage limit for the NiCo-hydroxide/NiO/CuO/Cu//AC/SS RAB is 1.5 V. However, from the three-electrode measurements, the maximum operating voltage window for the proposed RAB is 1.6 V. To maintain the long-term cycling stability and reversibility of the electrodes, we selected the voltage limit of 0–1.5 V [44, 45]. Figure 4c shows the CV curves for the NiCo-hydroxide/NiO/CuO/Cu//AC/SS RAB at various scanning rates within the voltage limit of 0–1.5 V. The CV curves maintain their CV shape at both lower and higher scan rates, suggesting better electrochemical features with excellent rate capability. The assembled NiCo-hydroxide/NiO/CuO/Cu//AC/SS RABs work very well, even at a high scan rate of 100 mV s^−1^ by sustaining the original CV curve, indicating the higher electrical conductivity of the electrode and electrolyte. Figure 4d presents the CD curves for the NiCo-hydroxide/NiO/CuO/Cu//AC/SS RAB at various currents within the voltage window of 0–1.5 V. Based on the CD curves, we calculated the specific capacitance and specific capacity for the NiCo-hydroxide/NiO/CuO/Cu//AC/SS RAB at various currents, and the corresponding results are presented in Fig. 4d. At the low current of 10 mA, the NiCo-hydroxide/NiO/CuO/Cu//AC/SS RAB shows a specific capacity of 219 mAh g^−1^ (263 F g^−1^), which then decreases to 207 mAh g^−1^ (249 F g^−1^) at a high current density of 20 mA, corresponding to a rate capability of 94.17%. Furthermore, the energy density and power density were calculated for the NiCo-hydroxide/NiO/CuO/Cu//AC/SS RAB by considering the discharge curves, and the corresponding results are plotted in the Ragone plot (Fig. 4f). The device exhibited the highest energy density of 82.42 Wh kg^−1^ at a power density of 1630.43 W kg^−1^, and a high power density of 3660.87 W kg^−1^ at an energy density of 77.89 Wh kg^−1^. Importantly, the assembled NiCo-hydroxide/NiO/CuO/Cu//AC/SS RAB simultaneously shows higher energy and power densities, thereby expanding the applicability of the battery technology. Further, the obtained energy densities are higher than those for lead acid, vanadium redox flow, and aqueous LIBs and SIBs [46, 47]. EIS was further used to investigate the electrochemical properties of the NiCo-hydroxide/NiO/CuO/Cu//AC/SS RAB. The Nyquist plot (Fig. 4g) shows that *R*_s_ and *R*_ct_ are less than 3 Ω, indicating the occurrence of facial electrochemical reactions between the electrode material and electrolyte ions. In addition, long-term cycling stability measurements were carried out for the NiCo-hydroxide/NiO/CuO/Cu//AC/SS RAB, and the corresponding results are presented in Fig. 4h, i. As shown in Fig. 4h, the NiCo-hydroxide/NiO/CuO/Cu//AC/SS RAB shows excellent cycling stability by maintaining 94% of capacity over the 5000 CD cycles. Moreover, the NiCo-hydroxide/NiO/CuO/Cu//AC/SS RAB shows a better columbic efficiency of 93%, even after 5000 cycles, representing the excellent reversibility of the electrode materials. Moreover, Fig. 4i shows the initial and final CD cycles with similar electrochemical characteristics, indicating the superior electrochemical performance of the NiCo-hydroxide/NiO/CuO/Cu//AC/SS RAB. The above-mentioned results indicate that the dendritic-structured NiCo-hydroxide/NiO/CuO/Cu electrode exhibits excellent electrochemical features that are suitable for high-energy RAB applications.

## Conclusions

In conclusion, a uniform dendritic-structured NiCo-hydroxide/NiO/CuO/Cu electrode was successfully fabricated over a waste Cu wire via a low-cost and scalable process for a high-energy RAB. The electrochemical investigation for the developed RAB shows excellent features in terms of a high-energy-density (82.42 Wh kg^−1^), excellent specific capacity (219 mAh g^−1^), and long-term cycling stability (94% capacity retention over 5000 cycles). The excellent electrochemical properties result from the innovative design of the hierarchical nanostructure with a highly porous branched nanostructure that provides a multichannel for facial and fast ion transportation during the charge/discharge process. More importantly, the simple synthetic approach provided in this work is highly repeatable and easy to scale up for different applications. Finally, the presented approach and material meet the requirements of cost-effectiveness, abundance, and high effectiveness of the electrode for advanced eco-friendly energy storage devices.

## Electronic supplementary material

Below is the link to the electronic supplementary material.

Supplementary material 1 (PDF 319 kb)
