# Supplementary material for: Dendritic Nanostructured Waste Copper Wires for High-Energy Alkaline Battery
Source: Nanomicro Lett. 2019 Dec 12;12:1. doi: 10.1007/s40820-019-0337-2 (PMC7770717; doi:10.1007/s40820-019-0337-2)
Supplement: Supplementary file 1 — Supplementary material 1 (PDF 319 kb) [file 40820_2019_337_MOESM1_ESM.pdf]

Supporting Information for

Dendritic Nanostructure based on Waste Copper Wires for High-Energy Alkaline Battery

Nilesh R. Chodankar[1, 2], Su-Hyeon Ji[1], Young-Kyu Han[2], Do-Heyoung Kim[1, *]

[1]School of Chemical Engineering, Chonnam National University, Gwangju 500-757, South Korea

[2]Department of Energy & Materials Engineering, Dongguk University, Seoul, 100-715, Republic of Korea

*Corresponding author. E-mail: kdhh@chonnam.ac.kr (Do-Heyoung Kim)

Supplementary Figures

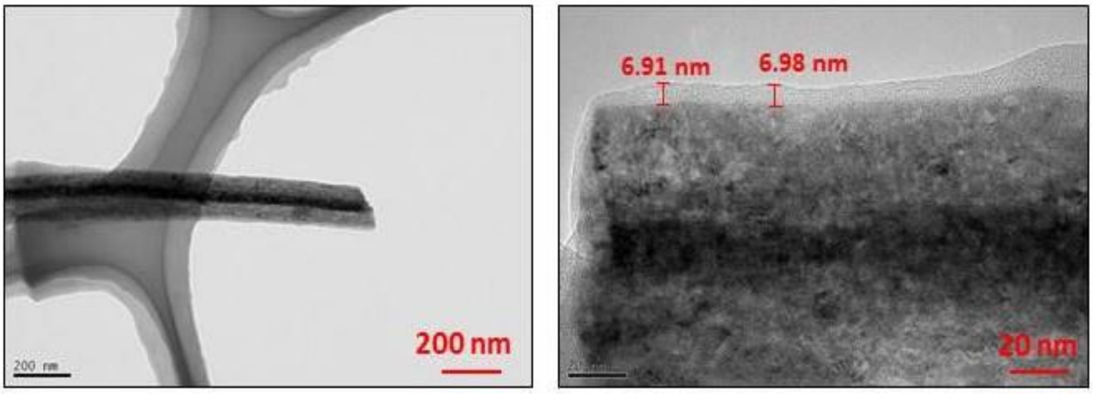

Fig. S1 TEM images for the NiO/CuO/Cu sample

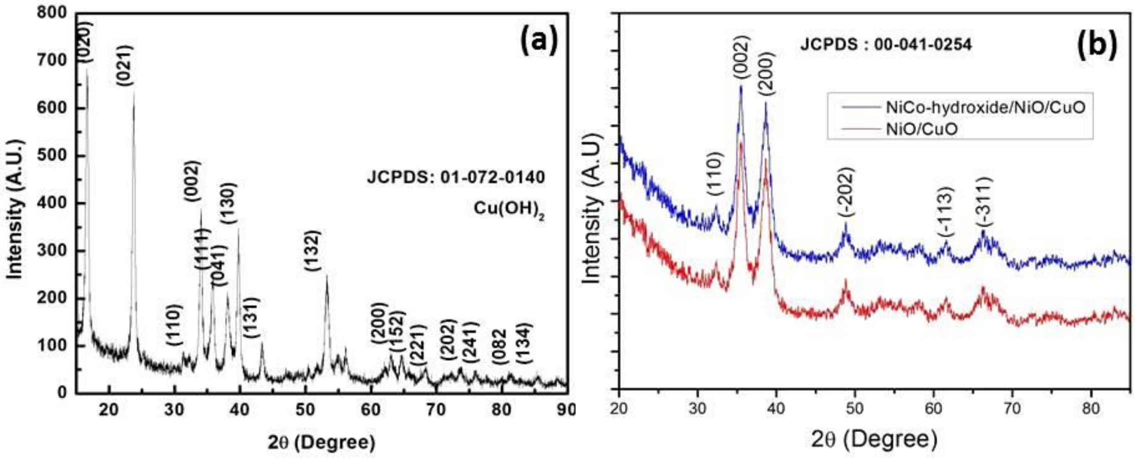

Fig. S2 XRD patterns of the prepared electrodes

The phase and structure of the prepared electrode were evaluated by XRD, and the corresponding results are presented in Fig. S2. The XRD spectra for the $Cu(OH)_2$/Cu electrode match the standard orthorhombic structure of $Cu(OH)_2$ well (Reference code: 01-072-0140).

Furthermore, the XRD spectra for the NiO/CuO/Cu and NiCo-hydroxide/NiO/CuO/Cu electrodes are presented in Fig. S2b. As observed in the XRD spectra, we were not able to find any reflection peaks for the NiO or NiCo-hydroxide material. As the thickness of the NiO ALD was less than 10 nm, it was very difficult to detect the reflection peaks for the XRD of NiO. However, the low process temperature for the NiCo-hydroxide did not result in any prominent peak in the XRD result. These results indicate the amorphous formation of the NiCo-hydroxide/NiO over the CuO/Cu wire. Furthermore, both XRD spectra are well-matched with the standard monoclinic CuO phase (Reference code: 00-041-0254).

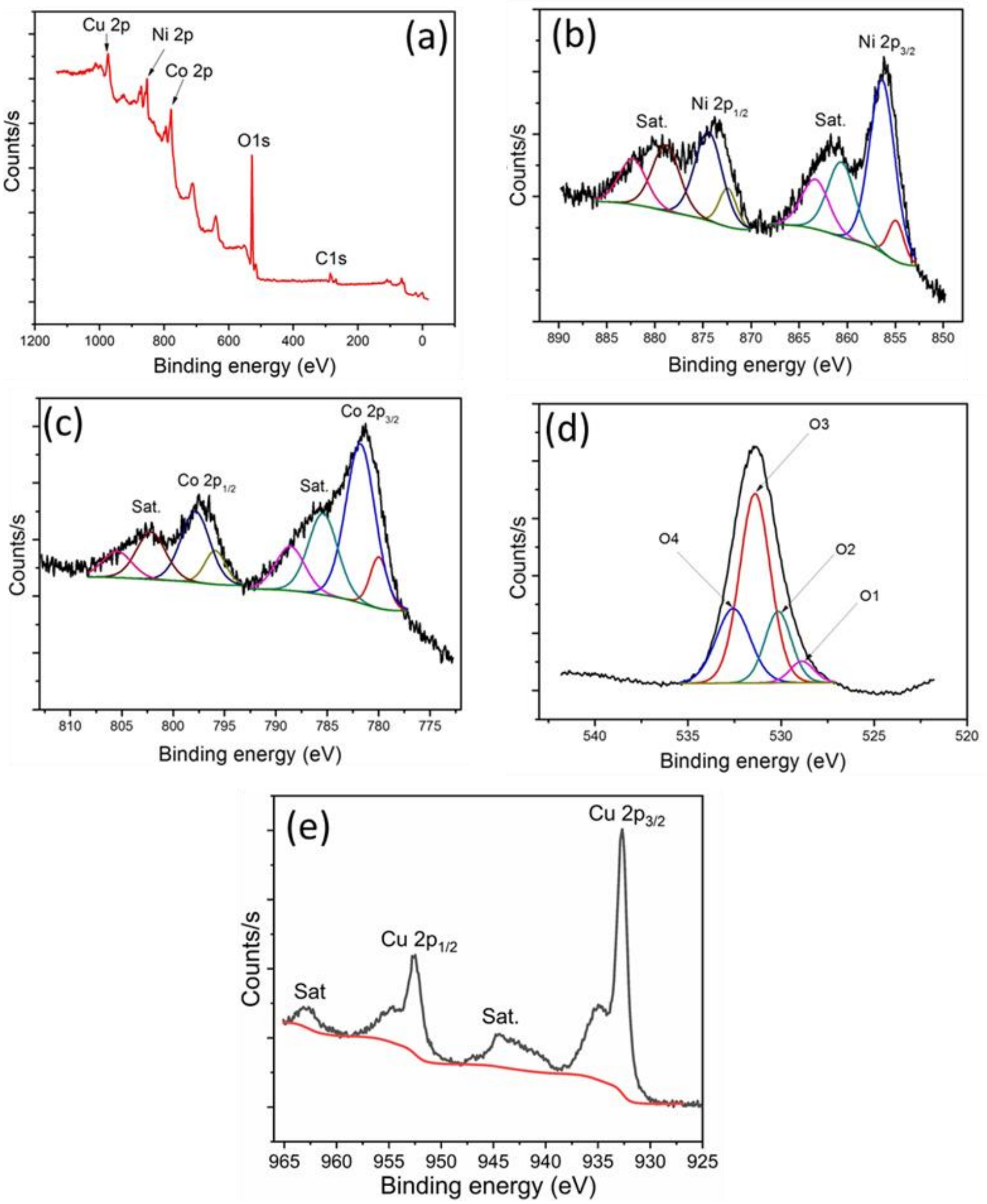

Fig. S3 (**a**) The broad scan XPS spectra for the NiCo-hydroxide/NiO/CuO/Cu electrode, the narrow scan XPS spectra for the (**b**) Ni 2p, (**c**) Co 2p, (**d**) O 1s, and (**e**) Cu 2p for the NiCo-hydroxide/NiO/CuO/Cu electrode

The surface chemical state for the NiCo-hydroxide/NiO/CuO/Cu electrode was studied by XPS analysis. Figure S3a shows the survey scan XPS profile, which displays the prominent peaks for Ni 2p, Co 2p, O 1s, and C 1s, suggesting the existence of Ni, Co, O, and C elements on the

surface of the prepared electrode. In the XPS profile, we were not able to find any peak for Cu, as XPS only provides information about the surface of the sample (up to 10 nm). The Ni 2p and Co 2p XPS profiles were fitted using the Gaussian fitting method, and the corresponding results are presented in Fig. S3b, c. The narrow-scan XPS profiles for Ni 2p show the major peaks at binding energies of 870.5 and 853.05 eV, which are assigned to the Ni $2p_{1/2}$ and Ni $2p_{3/2}$, respectively, demonstrating the existence of both Ni^{2+} and Ni^{3+}, respectively. The strong peaks at 778.3 eV for Co $2p_{3/2}$ and 794.2 eV for Co $2p_{1/2}$ confirm the presence of both Co^{3+} and Co^{2+} in the NiCo-hydroxide/NiO/CuO/Cu electrode. The narrow-scan spectrum for O 1s is presented in Fig. 3d, which is split into four major contributions denoted as O1, O2, O3, and O4 at binding energies of 528.9, 530.3, 531.8, and 533.1 eV, respectively. The O1 contribution is associated with the typical metal–oxygen bond; the contributions for O2 and O3 are due to the oxygen in the hydroxyl groups, while that for O4 is related to the physio-/chemisorbed water at and within the surface.

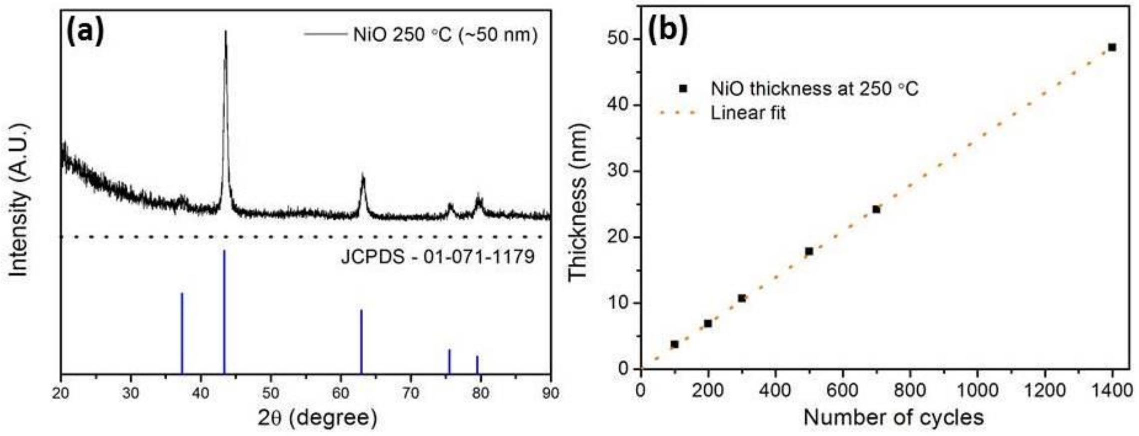

Fig. S4 (**a**) XRD spectrum for NiO prepared by atomic layer deposition over the silicon substrate for 50 nm at 250 °C and (**b**) thickness versus cycle number for NiO at a reaction temperature of 250 °C

For the sample NiO/CuO/Cu, we could not find any peak for NiO, as the thickness of NiO is less than 10 nm. Therefore, to confirm NiO formation via ALD, we prepared 50 nm ALD NiO over the silicon substrate and conducted GIXRD analysis. Figure S4a shows the XRD spectra for the 50 nm NiO prepared by ALD at a reaction temperature of 250 °C. The XRD data is well-matched with the standard NiO JCPDS card (01-071-1179), which confirms the formation of pure NiO. Moreover, to confirm the linearity of the ALD NiO process, the deposition of NiO was carried out for various cycles at a reaction temperature of 250 °C. As observed in Fig. S4b, there is a linear increment in the thickness of NiO with cycle number, suggesting the excellent linearity of the NiO ALD process. Here, the thickness of NiO over the silicon substrate was measured by ellipsometry (LSE Stokes ellipsometer, Gaertner Scientific, USA).
